# Genetic relatedness analysis reveals the cotransmission of genetically related *Plasmodium falciparum* parasites in Thiès, Senegal

**DOI:** 10.1186/s13073-017-0398-0

**Published:** 2017-01-24

**Authors:** Wesley Wong, Allison D. Griggs, Rachel F. Daniels, Stephen F. Schaffner, Daouda Ndiaye, Amy K. Bei, Awa B. Deme, Bronwyn MacInnis, Sarah K. Volkman, Daniel L. Hartl, Daniel E. Neafsey, Dyann F. Wirth

**Affiliations:** 1 Department of Immunology and Infectious Diseases, Harvard T. H. Chan School of Public Health, Boston, MA 02115 USA; 2 Broad Institute, Cambridge, MA 02142 USA; 3 Faculty of Medicine and Pharmacy, Cheikh Anta Diop University, Dakar, Senegal; 4 School of Nursing and Health Sciences, Simmons College, Boston, MA 02115 USA; 5 Department of Organismic and Evolutionary Biology, Harvard University, Cambridge, MA 02138 USA

**Keywords:** Malaria, Genomics, Epidemiology, Relatedness, Transmission, Coinfection, Polygenomic infection, Cotransmission, Superinfection

## Abstract

**Background:**

As public health interventions drive parasite populations to elimination, genetic epidemiology models that incorporate population genomics can be powerful tools for evaluating the effectiveness of continued intervention. However, current genetic epidemiology models may not accurately simulate the population genetic profile of parasite populations, particularly with regard to polygenomic (multi-strain) infections. Current epidemiology models simulate polygenomic infections via superinfection (multiple mosquito bites), despite growing evidence that cotransmission (a single mosquito bite) may contribute to polygenomic infections.

**Methods:**

Here, we quantified the relatedness of strains within 31 polygenomic infections collected from patients in Thiès, Senegal using a hidden Markov model to measure the proportion of the genome that is inferred to be identical by descent.

**Results:**

We found that polygenomic infections can be composed of highly related parasites and that superinfection models drastically underestimate the relatedness of strains within polygenomic infections.

**Conclusions:**

Our findings suggest that cotransmission is a major contributor to polygenomic infections in Thiès, Senegal. The incorporation of cotransmission into existing genetic epidemiology models may enhance our ability to characterize and predict changes in population structure associated with reduced transmission intensities and the emergence of important phenotypes like drug resistance that threaten to undermine malaria elimination activities.

**Electronic supplementary material:**

The online version of this article (doi:10.1186/s13073-017-0398-0) contains supplementary material, which is available to authorized users.

## Background

The recent push for malaria eradication highlights a growing need to accurately monitor changes in malaria transmission and assess the impact of interventions. Population genomic analyses and genetic epidemiology models can be powerful tools for monitoring declining transmission rates and evaluating the efficacy of public health interventions. Metrics of population genetic structure have been used to characterize parasite populations in low transmission regions [1–4] and, in combination with epidemiological modeling, to monitor changes in transmission rate [5].

Previous studies have largely relied on the sequences obtained from monogenomic (single-strain) infections, which may not provide an accurate representation of the genetic structure within the population. Polygenomic (multiple-genome) infections exhibit reduced genetic diversity relative to the total genetic diversity of all strains in the local population [6] and are known to be composed of genetically similar parasite strains [7–10], regardless of the genetic markers used. Understanding how polygenomic infections are formed, and incorporating the consequences of these infections on transmission patterns into genetic epidemiology models would help improve monitoring and evaluating systems within malaria elimination programs.

Historically, the formation of polygenomic infections has been assumed to be a function of the entomological inoculation rate (EIR), or the number of infectious bites per human per day [11] because multiple mosquito bites greatly enhance the probability of independent infections within a single human host from multiple mosquitoes (superinfection). Current epidemiology models largely operate under the assumptions of superinfection [12–14], which has been supported by the increased incidence of polygenomic infections in high transmission areas [15]. In high transmission areas, patients are exposed to numerous infectious bites, thus raising the chance of superinfection and the creation of new polygenomic infections. Under superinfection, strains within polygenomic infections are randomly and independently sampled from the local population.

The assumption of superinfection in epidemiology models is at odds with the observed similarity of strains within polygenomic infections [7–10], because superinfection cannot easily account for the high degree of similarity between strains within polygenomic infections. Relatedness among genomes in polygenomic infections is commonly attributed to cotransmission, or the simultaneous transfer of multiple, distinct parasite genomes from a single mosquito bite. Because the parasite undergoes sexual reproduction within the mosquito vector, cotransmitted parasites are expected to be genetically related to one another [8]. After a single cotransmission event, cotransmitted infections may be composed of F_1_ hybrids as well as unrecombined parental genomes. Subsequent cotransmission events (serial cotransmission) may result in high degrees of relatedness within polygenomic infections. Serial cotransmission chains constrain parasites to mating with their relatives, resulting in a steady increase in the average relatedness between cotransmitted strains. Extremely high degrees of genetic relatedness have been proposed to be signatures of serial cotransmission events that could be used to identify infections due to serial cotransmission [8].

Determining whether current epidemiological models can realistically simulate the relatedness within polygenomic infections is of key public health interest when these models use population genomics to monitor declining transmission rates. Here, we quantified the genetic relatedness of genomes within individual polygenomic infections using a hidden Markov model (HMM) to measure the proportion of the genome that is inferred to be identical by descent (IBD). Our HMM allows us to distinguish regions of the genome that are more likely to be identical due to random chance and population structure from regions of the genome that are more likely to be identical due to shared inheritance. These IBD estimates were compared to the relatedness expected with superinfection, which was simulated as the random sampling of parasites from Thiès, Senegal, which was represented by 146 monogenomic infections previously collected from the region.

Our polygenomic infections comprised 31 infections collected from patients in Thiès, Senegal in the years 2011–2013. Thiès lies 70 km away from the capital city of Dakar, a hypoendemic region with an EIR <5 [16]. In 2005, Senegal implemented a redesigned National Malaria Control Programme (NMCP) aimed at improving insecticide-treated mosquito net coverage, indoor residual spraying coverage, preventative treatment coverage for pregnant women and children under five, and antimalarial treatment coverage. Since then, there has been a significant decrease in the number of confirmed cases, going from 1,555,000 cases in 2006 to 174,000 cases in 2009 [17]. As of 2009, the prevalence in Thiès was ~3% [17] and has since fallen further.

Our findings indicate that cotransmission is common in Thiès, Senegal, and that genetic epidemiology models can be made to more accurately reflect relatedness within polygenomic infections by incorporating cotransmission. These findings have important implications for the application and use of genetic tools to understand malaria transmission dynamics, to assess the impact of malaria elimination interventions, and to study the consequences of these interventions on potentially undermining traits such as drug resistance emergence.

## Methods

### Sample and sequence collection

All patient samples were collected at clinics located in three different areas of Senegal: Thiès, Pikine, and Velingara. These samples were collected between approximately September and December each year, which roughly corresponds to the period just following the rainy season in Senegal. Participants reporting acute fevers and suspected of being infected with malaria (e.g., mild uncomplicated malaria infection) with no reported history of antimalarial therapy were considered for inclusion in our study. Participants were diagnosed for malaria based on microscopy and rapid diagnostic tests. Samples were anonymous and coded as to country (Senegal or Sen), collection village (T = Thiès, P = Pikine, V = Velingara), and sample number collected from the clinic (001 to 999), and were also identified by year (e.g., 2011 or 11) to create, e.g., a sample number of ”SenT009.11,” which was collected from Thiès, Senegal in the year 2011 and represents the ninth sample (009) collected that year.

We sequenced 190 *Plasmodium falciparum* genomes from patient-derived material collected from Senegal, of which 176 were collected from Thiès, 4 from Velingara, and 10 from Pikine. These samples were initially identified as monogenomic using a 24-single nucleotide polymorphism (SNP) molecular barcode [18]. Barcodes were genotyped using a high-resolution melting (HRM)-based assay [2, 18]. The parasite strains were culture adapted at the Harvard T.H. Chan School of Public Health and sequenced at the Broad Institute using Illumina Hi-Seq (Illumina, Inc., San Diego, CA, USA) machines.

We also sequenced a set of 111 samples collected exclusively from Thiès, Senegal during the years 2011–2013. Unlike our previously mentioned samples, genomic DNA was extracted directly from patient samples to avoid strain ascertainment bias and the potential loss of low frequency strains. Genomic DNA was extracted using a QiAmp DNA Blood Mini kit (Qiagen, Valencia, CA, USA) according to manufacturer’s specifications. These samples were sequenced at the Broad Institute using Illumina Hi-Seq machines.

Sequencing reads were aligned using the Burrows-Wheeler Aligner (version 0.5.9-r16) [19] against the 3D7 reference assembly (PlasmoDBv7.1) [20] to create BAM files. Variant calls and consensus sequences for each sample was determined using GATK Unified Genotyper [21]. A full list of the individual parameter and quality-score thresholds can be found in the supplementary information of [5].

### Defining our monogenomic infection dataset

To determine the expected relatedness of superinfection, we needed to identify a set of monogenomic infections to represent the parasites present in Thiès, Senegal. To do this, we relied on a set of 190 samples that were previously sequenced and identified as monogenomic using a 24-SNP barcode. For this study, we decided to use stricter criteria to identify monogenomic samples. Within each of the 190 sequences classified as monogenomic by barcode, all sites with a non-unanimous read pileup were first identified, resulting in 1.1 million variant positions. These positions were then filtered to have a read depth of at least 10 across 90% of the samples, to be strictly biallelic, and to be found in at least 2 of the 190 samples. A preliminary set of 440,000 SNPs passed these criteria, which were then used to reclassify each of the 190 putatively monogenomic samples. Monogenomic samples were reclassified by calculating the proportion of the 440,000 sites with a unanimous read support within each of the 190 samples. Those samples where the proportion was 80% or higher were considered monogenomic, which identifed 146 monogenomic samples, all of which originated from Thiès. The read pileups of these samples over the preliminary set of 440,000 SNPs have less than 0.0005% non-unanimous reads (Additional file 1: Figure S1).

Because our set of 440,000 SNPs was derived using information from all 190 samples, which could represent a mix of monogenomic and potentially cryptic polygenomic samples, we chose a more stringent set of SNPs based solely on the information drawn from monogenomic samples. Of the 146 monogenomic samples, 56 were randomly chosen to further filter our set of 440,000 preliminary SNPs. Sites where the read pileup across all 56 samples was less than or equal to 0.01% or those that lacked reads in more than 1 of the 56 samples were also removed. After applying these filters, we identified a set of 3132 SNP positions that were used to analyze the genetic relatedness within polygenomic infections.

### Defining our final polygenomic infection dataset

These 3132 SNPs were then used to identify polygenomic infections from the set of 111 samples collected from Senegal during the years 2011–2013. Samples where less than 30% of the 3132 SNPs had at least one read were excluded from our analysis, leaving us with 31 polygenomic infections. For each of the remaining samples, we removed sites that were supported by a single read. All samples in which at least 95% of the remaining sites were completely unanimous were classified as monogenomic, while any sample with a proportion less than 95% was classified as polygenomic.

### Estimating relatedness using a hidden Markov model

For each sample, we calculated relatedness between sample pairs by first identifying regions of the genome that are inferred to be IBD based on the likelihood of observing identity due to random chance using a hidden Markov model (HMM) [5]. The model has two hidden states: IBD, inherited from the same ancestor, or different by descent (DBD), inherited from different ancestors. Sequence pairs are reduced to a series of discordant and concordant calls, depending on the observations made at each SNP site. Sites where both sequences have the same allele are considered concordant, while sites where each sequence has a different allele are considered discordant. The model then calculates the probability of observing concordant or discordant genotypes under the assumption of IBD or DBD by using the population allele frequencies at that site, the error rate, and the probability of transitioning from one hidden state to the other. The probability of transitioning from IBD to DBD between two SNPs is proportional to the physical distance between them and is influenced by the overall recombination rate. The HMM then uses a Viterbi algorithm to identify the most probable path of hidden states. An overall estimate of relatedness for each comparison was obtained by summing the total proportion of the optimum path that is in IBD.

Delete-a-group jackknife analysis was performed to obtain jackknife estimates of the mean and jackknife estimates of the standard error of the mean. Groups were defined by dividing the genome into 10 mutually exclusive groups by scanning across the genome and placing the *i*th SNP into the *i*th group. After all 10 groups have at least one SNP, the process is repeated, placing the *i* + 10th SNP into the *i*th group, and continuing until the end of the genome. This effectively randomizes the SNPs in each group and ensures that the number of SNPs and distribution of SNP locations within each group is evenly distributed.

### Generating artificial mixed genome samples

Genomic DNA mixtures were generated by mixing DNA obtained from five distinct culture-adapted parasite strains (SenT148.09, SenT111.09, SenT165.09, SenT033.09, and SenT015.09) in proportions described in Additional file 1: Table S1. Genomic DNA was extracted from adapted parasite cultures using a QiAmp DNA Blood Mini kit (Qiagen, Valencia, CA) according to manufacturer specifications. DNA concentrations were determined by a NanoDrop Spectrophotometer (Thermo Fisher Scientific) and a barcode-based quantification assay [18]. Each mixture had a total DNA concentration of 5 ng/μl.

### Constructing pseudohaplotypes

Pseudohaplotypes were constructed by examining the read pileups at each of the available 3132 SNPs for each polygenomic infection. Sites were categorized into heterozygous sites, a site where at least one read had an alternate allele, and homozygous sites, a site where all the reads had the same allele. Pseudohaplotypes were constructed by randomly assigning the allelic states of each site to one of two constructed haplotypes. For homozygous sites, both haplotypes received the same allelic state. For heterozygous sites, one haplotype received the major allelic state (the allele with the greater read support), while the other haplotype received the minor allelic state (the allele with the lower read support). These pseudohaplotypes preserve the physical order and distance between each of the available 3132 trusted SNPs and the order of concordant and discordant calls, but do not establish true linkage phase.

### Generating subsets to test the limitations of the HMM

Subsets were generated by randomly choosing without replacement from the 3132 SNPs. The largest of these subsets contained 90% of the 3132 SNPs, while the smallest contained 10% of them. Each subset was repeated 40 times to obtain estimates of the mean and standard deviation.

### Calculating concordance

For each pairwise comparison, concordance was calculated as the number of sites with the same allelic identity divided by the number of sites examined. Due to the presence of missing data, the number of sites examined fluctuated. If a site was missing in one or both of the strains being compared, then the site was excluded from the analysis. In addition, sites where only the major allele was present were also excluded.

### Simulating expected relatedness under superinfection

Superinfection was simulated as a random sampling of parasites collected throughout Thiès, Senegal. We assumed that the parasite population in this region was completely mixed, with no heterogeneity in population structure or transmission intensity. The expected relatedness under the superinfection hypothesis was calculated by quantifying the relatedness between our set of 146 monogenomic infections.

To make the data from our simulation more comparable with the data obtained from our polygenomic infections, we generated a series of bootstrap resampled distributions of the mean relatedness. Simple random sampling bootstrap distributions were generated by randomly sampling 40,000 sets of 31 monogenomic pairs and calculating the average relatedness among these sample pairs. To create weighted bootstrap distributions, we extracted the barcode sequence from each of the monogenomic infection whole genome sequences and identified it with one of the barcode sequences within our 24-SNP barcode dataset. The identities of at least 22 of the 24 barcode positions needed to be identical to be considered the same sequence. The observed frequency of each 24-SNP barcode was used to infer the population frequency of the parasite strain within each monogenomic infection. A weighted bootstrap distribution of mean relatedness was created by calculating the randomly sampling 40,000 sets of 31 monogenomic infection pairs, where each pair was weighted according to the probability of drawing that particular sample pair.

The *p* values for each bootstrap distribution were calculated by counting the number of times our sample mean was greater than or equal to the observed mean relatedness in our 31 polygenomic infections (relatedness = 0.38).

### Identifying monogenomic infections that were related to polygenomic infections

For each polygenomic infection, we used the HMM to compare the observed within-polygenomic infection IBD segments with the corresponding genomic regions in each of the 146 monogenomic samples. Related monogenomic infections were identified as those that contributed a significant fraction of the polygenomic infection’s IBD segments.

## Results

### Relatedness within polygenomic infections

**Fig. 1:**
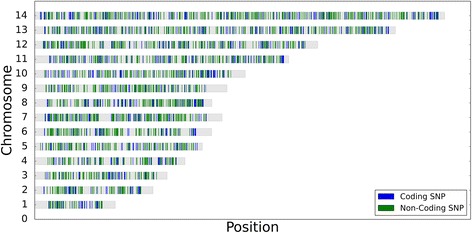
Trusted SNP set marker map. A representation of the *P. falciparum* genome and the location of each of the 3132 trusted SNPs. *Gray bars* represent individual chromosomes. *Blue lines* indicate the location of coding SNPs, and *green lines* represent the location of non-coding SNPs

To quantify the relatedness of strains within each infection, we identified a set of 3132 SNPs that had passed a set of read mapping filters designed to remove variant positions liable to yield erroneous heterozygous signals due to read mapping and/or base calling errors. These trusted SNPs form a sensitive panel for detecting heterozygous positions within polygenomic samples, and can be used to mark IBD segment boundaries (Fig. 1). The majority of our SNPs fall within coding regions (77% coding, 23% noncoding). The proportion of reads supporting the major allele at each of these sites reflects the expected ratio of individual strains in sets of mixtures created from genomic DNA to control for both genome diversity and relative proportions (Additional file 1: Figure S2).

We sequenced 111 polygenomic infections collected from patients in Senegal arriving at clinic for treatment for mild uncomplicated malaria infection during the years 2011–2013. Each sample had an average of 58 million reads, but because genomic DNA was extracted directly from patient material and not depleted of host material before sequencing, only 1% of them aligned to the *P. falciparum* genome. As a result, some of the polygenomic infections lacked coverage at all the trusted SNP locations. Samples where >30% of the trusted SNP sites lacked sequencing reads were excluded from our analysis, leaving us with a total of 31 polygenomic infections. For each of the remaining polygenomic infections, we excluded sites with <1 read from our analysis. After excluding these sites, we found that the range of usable sites per sample spanned from 300 to 3132 SNPs. Samples collected from 2011 had the highest mean number of usable sites (3113 sites), while samples collected in 2012 and 2013 had a lower mean number of usable sites (865 and 1172 sites, respectively) (Additional file 1: Figure S3). At sites where there were at least two reads, we found that the average read depth in our samples was 7.68; read depth in samples collected from 2011 was higher (12.74) and those collected from 2012 and 2013 had a lower read depth (3.08 and 3.62, respectively).

To quantify the relatedness, or proportion of the genome that is identical by descent (IBD), within each polygenomic infection, we used an HMM that was previously used to quantify the relatedness of genomes present in monogenomic infections collected in Senegal [5]. Because our HMM examines sequence pairs as a series of discordant and concordant calls, we constructed two pseudohaplotypes that preserve the order and position of discordant and concordant calls to represent the genetic similarity of genomes within each infection. We use the term pseudohaplotype because the inferred haplotype does not necessarily establish the true linkage phase of haplotypes within polygenomic infections. These pseudohaplotypes are actually conservative representations of genetic similarity because they underestimate the true similarity between genomes when the polygenomic infection is composed of more than two strains. During the sampling timeframe and setting in Thiès, Senegal, the average complexity of infection (COI) in polygenomic infections is two [22], and the pseudohaplotypes reflect the genetic similarity of the genomes.

We first ran tests to determine if the variation in number of assayable SNPs would affect our estimates of relatedness. We calculated the relatedness between 27 monogenomic sample pairs using different numbers of SNPs taken from the complete set of 3132 SNPs. We found that the HMM is robust to differences in SNP number and that estimates of relatedness based on as few as 313 SNPs will consistently provide the same estimate as those based either on 3132 SNPs or an even larger set of 14,972 SNPs with a minor allele frequency of ≥0.05 among the samples from Senegal (Additional file 1: Figures S4 and S5).

**Fig. 2:**
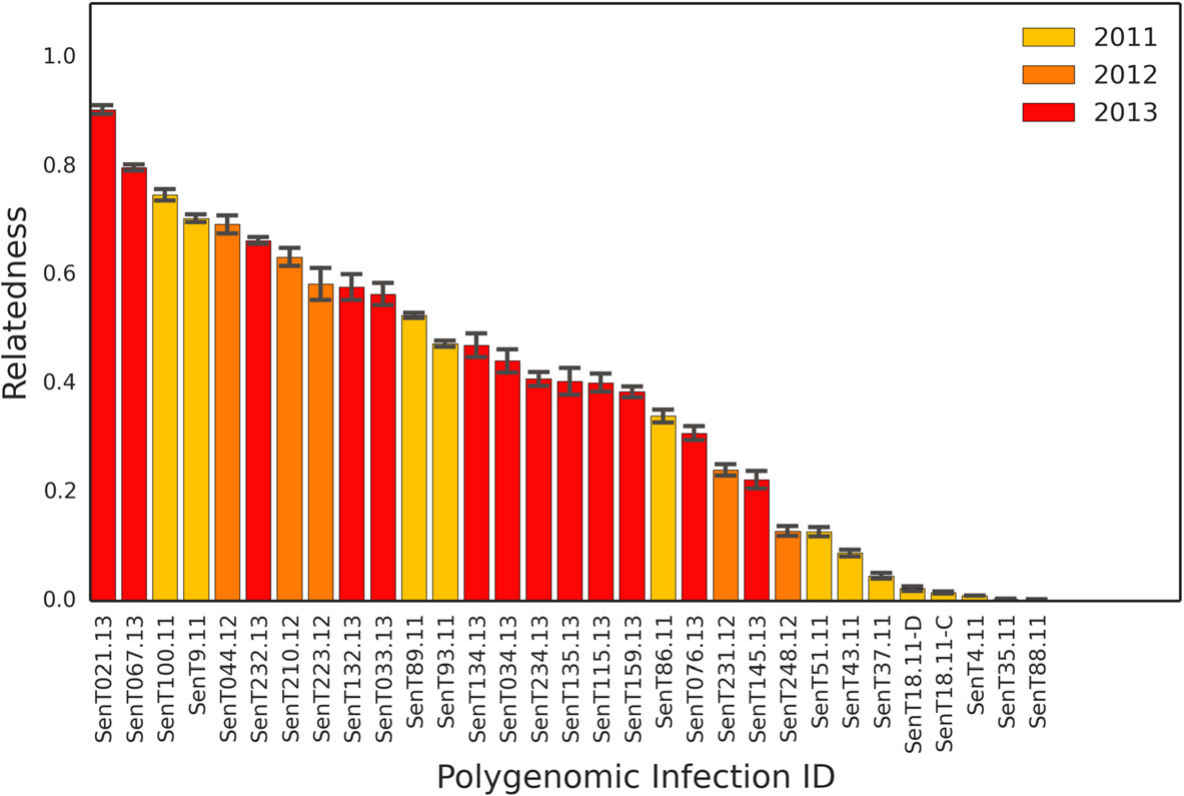
Relatedness within polygenomic infections. Barplots of jackknife estimates of the mean relatedness within 31 polygenomic infections collected from Senegal from 2011–2013. Error bars represent one jackknife estimate of the standard error of the mean. Relatedness is defined as the proportion of genome shared IBD between the strains comprising each polygenomic infection. While there is no clustering of relatedness by year, samples collected in 2011 are less related (average relatedness = 0.24) than samples collected in 2012 and 2013 (average relatedness = 0.46 and 0.50, respectively) (*p* value = 0.048, one-way ANOVA). Samples collected from 2012 and 2013 had lower coverage than those in 2011, which may contribute to their higher relatedness values

**Fig. 3:**
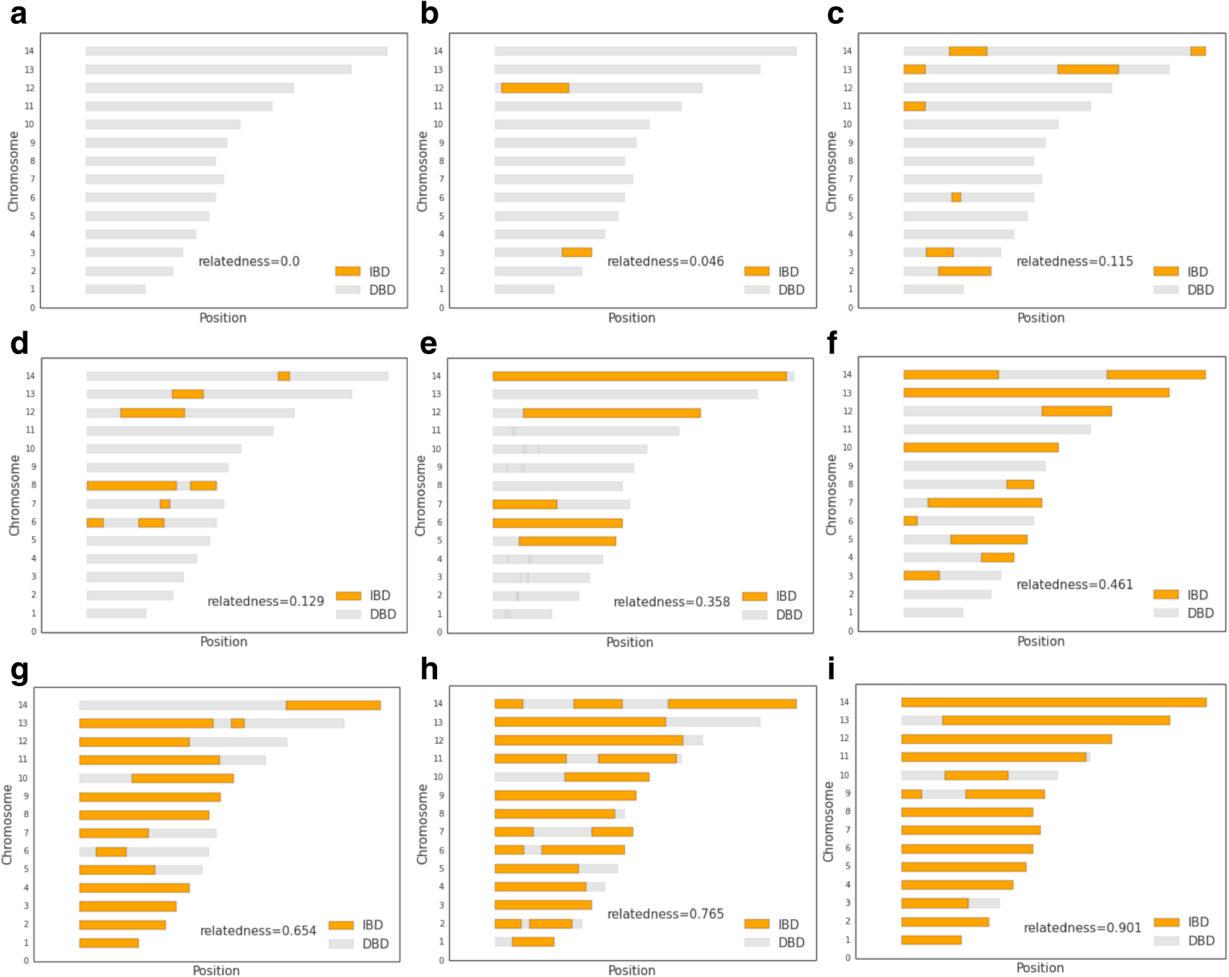
Polygenomic infection IBD maps. Representative IBD maps of nine different polygenomic infections. *Gray bars* represent sections of the genome that are not IBD among the strains present within the polygenomic infections. *Orange sections* represent regions of the genome that are IBD. **a** = SenT88.11, **b** = SenT37.11, **c** = SenT51.11, **d** = SenT248.12, **e** = SenT223.12, **f** = SenT093.11, **g** = SenT232.13, **h** = SenT100.11, **i** = SenT021.13

**Fig. 4:**
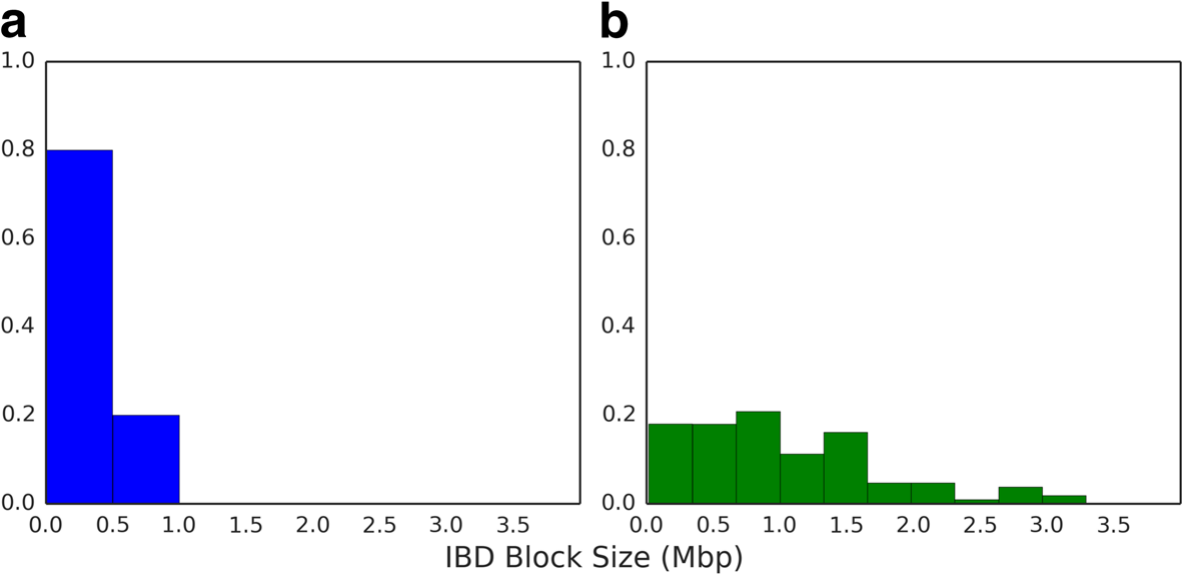
IBD block distributions within polygenomic infections. Distribution of IBD block sizes in megabase pairs (*Mbp*). IBD blocks were defined as contiguous segments of the genome that are IBD and are longer in highly related polygenomic infections (*p* value = 2.70 × 10^−8^, Mann–Whitney U). **a** Distribution of IBD block size in less related polygenomic infections (relatedness <0.30). Average block size is 0.31 Mbp with a standard deviation of 0.21 Mbp. **b** Distribution of IBD block sizes in highly related polygenomic infections (relatedness >0.30). Average block size is 1.04 Mbp with a standard deviation of 0.73 Mbp

We found that the estimated genetic relatedness within the 31 polygenomic infections is evenly distributed, ranging from completely unrelated (relatedness = 0.0) to highly related (relatedness = 0.90) (Fig. 2, Additional file 1: Figure S6). Across all years, we found that the average relatedness within a polygenomic infection was 0.38. To examine the distribution of IBD block sizes within each infection, we mapped each IBD block to its corresponding location in the *P. falciparum* genome (Fig. 3). There was a trend in genetic relatedness and IBD block size. Across all samples, the average IBD block size within the 31 polygenomic infections was 0.92 Mbp. After dividing infections into highly related infections, which were defined as having a relatedness of ≥0.30 (a value exceeding that expected of half- siblings, 0.25, but allowing for some uncertainty in the accuracy of our HMM) and less related infections (relatedness <0.30), we found that the average IBD block size among highly related infections was significantly longer (*p* value = 2.70 × 10^−8^, Mann–Whitney U). IBD blocks among highly related parasites (average IBD block size = 1.05 Mbp) were on average 0.73 Mbp longer than the block sizes across less related parasites (average IBD block size = 0.32 Mbp) (Fig. 4).

**Fig. 5:**
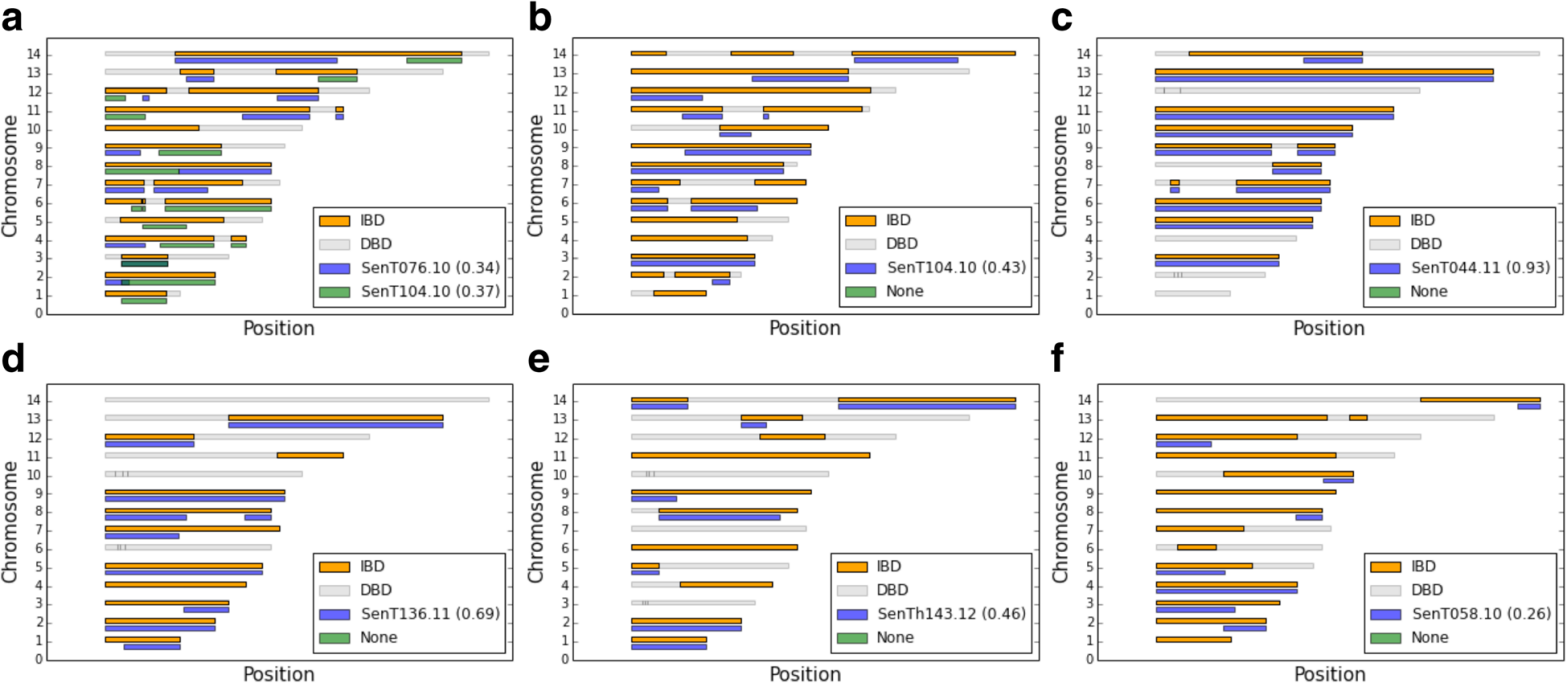
IBD maps within polygenomic infections and between monogenomic infections. Each subplot represents an individual polygenomic infection. **a** = SenT009.11, **b** = SenT100.11, **c** = SenT044.12, **d** = SenT210.12, **e** = SenT232.13, **f** = SenT232.13. *Orange/gray color scheme* represents the IBD map of the polygenomic infection, with *orange* representing regions of the genome that are IBD and *gray* representing regions of the genome not IBD. *Blue/green color schemes* represent regions of the genome that are IBD between the strains found within the polygenomic infections and a related monogenomic strain. *Blue bars* indicate that region of the genome is IBD with one of the monogenomic strains, while *green bars* indicate that region of the genome is IBD with the other monogenomic strain. *Values in parentheses* indicate the proportion of the within-polygenomic infection IBD block that is explained by a particular monogenomic infection

We also found that some of these polygenomic infections were related to parasite strains independently sampled from within the local population. We used the within-polygenomic IBD segment boundaries to generate IBD maps between the strains within polygenomic infections to the strains from monogenomic infections (Fig. 5). IBD segments create localized regions of the genome where the phase is known, allowing us to compare the strains from polygenomic infections to strains from the local population. For each of the polygenomic samples, we determined whether there were monogenomic samples sharing IBD segments with those within polygenomic infections and identified monogenomic samples that shared a large fraction of IBD with the within-polygenomic IBD segments.

For one polygenomic infection collected in 2011, SenT009.11, we identified two related strains, both of which were collected in the previous year (2010) among monogenomic infections. In the case of SenT009.11, the monogenomic samples SenT076.10 and SenT104.10 collectively shared IBD with 71% of the within-polygenomic IBD segments, contributing 33% and 36% of shared IBD, respectively. In this case, SenT076.10 and SenT104.10 each contributed to approximately half of the identifiable within-polygenomic IBD segments, with little overlap in the ancestral IBD segments. We also found that the relatedness between SenT076.10 and SenT104.10 was negligible (relatedness = 0.01) (Additional file 1: Figure S7), which could suggest that SenT009.11 is the result of a natural genetic cross between SenT076.10 and SenT104.10.

For five other polygenomic infections, we could identify one strain that was highly related to an independent monogenomic infection. The proportion of shared IBD blocks between each polygenomic infection and related monogenomic infection varied but was on average 0.51. One polygenomic infection shared an unusually large proportion of its IBD segments with its related monogenomic infection, where 93% of its IBD segments were with SenT044.11.

### Expected relatedness with superinfection

Under the superinfection hypothesis, polygenomic infections are composed of parasite strains sampled from the local population. Here, we simulated the formation of polygenomic infections through superinfection by sampling from a set of 146 monogenomic infections previously collected from Senegal around the same time and place as our 31 polygenomic samples. These samples exhibit negligible population structure [23]. A polygenomic infection was simulated by drawing two random sets of SNPs from the full set of 3132, where each set of SNPs represents one of a pair of genomes in a superinfection. We assumed pairs of genomes because the average number of unique strains in our sample of polygenomic infections is two [22].

Our first sampling scheme did not correct for either differences in sample size or any potential bias in the monogenomic samples. We created a naive simulation of superinfection by quantifying the relatedness between all possible 146-choose-2 monogenomic sample pairs. We found that the distribution of relatedness is positively skewed, with 99% of the comparisons having a relatedness of 0. Under this naive simulation, the average relatedness of simulated polygenomic infections under superinfection is only 0.007 (Additional file 1: Figure S8).

**Fig. 6:**
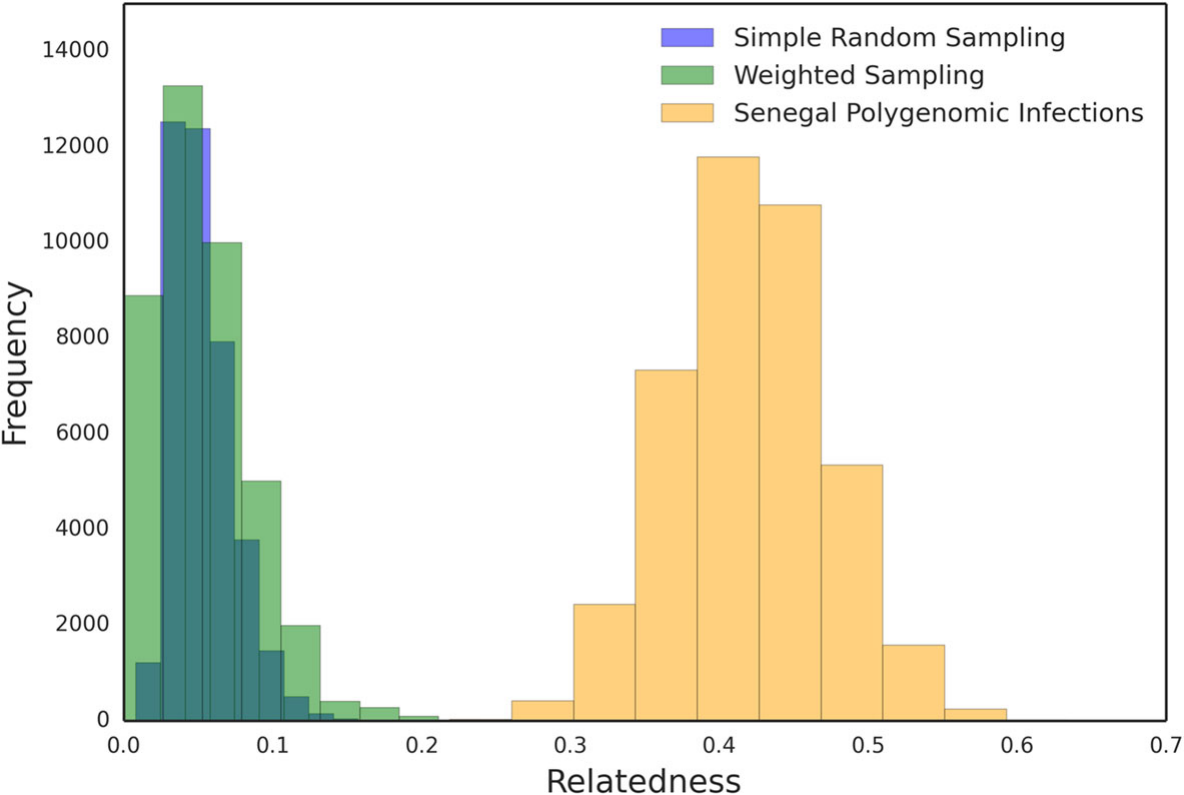
Expected relatedness under superinfection. Bootstrap distributions for the expected relatedness under superinfection were generated by randomly sampling with replacement 31 monogenomic pairs. For each set of 31 monogenomic pairs, we calculated the average relatedness and repeated this process 40,000 times to generate bootstrapped distributions of the mean relatedness between monogenomic infection pairs. Superinfection was simulated with either a simple random sampling scheme (*blue*), in which all sample pairs were equally likely, or a weighted sampling scheme (*green*), which uses the barcode frequencies of the corresponding monogenomic samples to weigh each sample pair. Bootstrap resampled distributions of expected relatedness in polygenomic infections are shown in *orange. p* values for both sampling schemes were ≤2.5 × 10^−5^

Because the distribution of relatedness within real polygenomic infections was based on only 31 samples, we wanted to generate a simulation that took into account sampling variation. To do this, we generated simple random sampling bootstrap distributions of the mean relatedness between sample pairs (Fig. 6, blue). We calculated the mean relatedness of 31 randomly chosen sample pairs and repeated this process 40,000 times. We found that the mean relatedness of this distribution was extremely low (0.02). In addition, to correct for any potential strain bias in the set of 146 monogenomic samples, we also generated a weighted bootstrap distribution where monogenomic sample pairs were weighed according to the frequency of the corresponding 24-SNP barcode for each strain (Fig. 6, green). The 24-SNP barcode consists of 24 putatively neutral, unlinked sites that were used to profile parasite diversity in Senegal [4]. After correcting for potential ascertainment bias that would lead to an underestimate of true relatedness among monogenomic samples in the population, we found that the expected relatedness under superinfection was still very low (0.048.)

However analyzed, the simulated superinfections severely underestimate the level of relatedness within polygenomic infections (*p* value in the naïve simulations = 1.1 × 10^−21^, Mann–Whitney U). Attempts to correct for sample size and strain bias failed to recapitulate the level of relatedness actually observed within polygenomic infections. In both bootstrap simulations, the relatedness within simulated superinfections is significantly lower than the relatedness observed within polygenomic infections, with *p* values ≤2.5 × 10^−5^ for both (*p* value calculated using resampling techniques).

## Discussion

Understanding the genomic composition of polygenomic infections is crucial for the assessment of transmission based on the genetic profile of malaria infections and for generating epidemiological models relating population genomics to transmission intensity. In this study, we investigated whether polygenomic infections simulated under superinfection conditions would accurately recapitulate the genetic relatedness observed in 31 natural polygenomic infections collected from patients in Thiès, Senegal. We first developed a strategy that offers a simple, cost-effective way of quantifying the relatedness within polygenomic infections without serial dilution or flow sorting single cells. Previous studies have characterized the relatedness within polygenomic infections by isolating individual parasite haplotypes through culture adaptation, serial dilution, or flow sorting [7–9]. Our pipeline uses standard Illumina sequencing reads to interpret the relatedness within polygenomic infections from direct patient samples without needing to establish linkage phase, which greatly increases the number of polygenomic infections one can examine. This approach trades the resolution of previous approaches in exchange for reduced sample preparation requirements and does not require that cells be preserved intact. Our methodology is more applicable to a broader range of samples, which may be useful for understanding the relatedness of polygenomic infections in different transmission settings.

However, alternative sequencing approaches should be considered when analyzing polygenomic infections with a COI > 2. While our approach works well when COI is 2, it underestimates the relatedness of polygenomic infections with COI > 2, since the constructed pseudohaplotypes will combine the differences across all strains in the infection. Polygenomic infections identified as being composed of apparently unrelated parasites by our method may in fact be composed of 3 or more strains of varying degrees of relatedness. Thus, the genomic haplotypes of more complex polygenomic infections should be established prior to using our HMM. Haplotypes can be established using sequencing technologies that generate longer reads, but haplotype reconstruction can be computationally challenging, especially in situations where the relative frequencies of strains are not the same (reviewed in [24]). Single-cell sequencing, which was previously used to calculate the relatedness of strains in polygenomic infections for both *P. falciparum* and *P. vivax* [9], has the advantage of avoiding complex haplotype reconstruction algorithms but is extremely labor intensive. Although our HMM will be useful for quantifying the relatedness of more complex infections, quantifying the relatedness of more complex polygenomic infections will require more sophisticated sequencing technologies or haplotype reconstruction algorithms.

Our study also contributes to a growing body of evidence indicating that cotransmission is common in natural parasite populations. Studies in low transmission areas, such as the Peruvian Amazon [10] and the Thai-Burma border [3, 8, 9], have reported highly related parasite strains within polygenomic infections. Highly related polygenomic infections are also observed in high transmission areas [7, 8], despite the fact that patients are exposed to large numbers of infectious mosquito bites. Here, we simulated superinfection as the random sampling of parasites from those found in Thiès, Senegal and found that a pure superinfection model fails to explain the observed relatedness within natural polygenomic infections.

When constructing our superinfection simulations, we assumed that the parasite population in Thiès, Senegal was completely mixed, with no hidden population structure. This is an oversimplification, since malaria transmission becomes clustered around transmission foci at low transmission settings [25]. To date, there is no genetic evidence of population structure in this region [23], but this could be because the sample collection was insufficient to capture the effects of localized transmission foci or other spatial heterogeneity effects. Spatial clustering can result in localized inbreeding events that raise the relatedness of parasites in the surrounding region and thus increase the relatedness of true superinfections. We believe it is unlikely that the relatedness in our polygenomic infections is due solely to the sampling of infections from transmission clusters, since the majority of parasites in Senegal are unrelated to one another [5] and because patients reporting to clinic do not necessarily live in the same areas of Thiès. However, since patient data regarding residence and travel history were not made available, we cannot exclude this possibility. We recognize that the relatedness of superinfection events could be influenced by the inhibition of future strains due to the host immune response, but we suspect these are effects are small, and previous studies have observed similar findings in children with little or no premunition [8].

The wide range of polygenomic relatedness values in Senegal suggests that our polygenomic infections may represent a mix of both superinfection and cotransmission events. Some polygenomic infections include apparently unrelated parasite genomes, but it is unclear whether these result from superinfection or the cotransmission of unrecombined parasite genomes. With self-fertilization in the mosquito, it is theoretically possible for two unrelated genomes to be cotransmitted by a single mosquito host. This problem could be exacerbated if there is a preference for self-fertilization or selection occurring within the mosquito vector and human host. These complications make it difficult to estimate the rate of cotransmission based solely on the frequency of highly related genomes in polygenomic infections. Nonetheless, our data suggest that cotransmission is frequent in Thiès, Senegal and may be a dominant mechanism by which polygenomic infections persist in low transmission settings.

Previously, Nkhoma et al. [8] suggested that extreme degrees of genetic relatedness within polygenomic infections could be the result of repeated cotransmission events, or serial cotransmission chains. Analyses of experimental crosses indicate that the mean relatedness between F_1_ progeny is approximately normally distributed with a mean of 0.52 and a standard deviation of 0.08 [26]. In our data (Fig. 2), 6.5% of polygenomic infections exhibit genomic relatedness exceeding 0.76, which is three standard deviations above the mean in experimental crosses, and also suggests serial cotransmission. The relatively low frequency of such closely related genomes might suggest that serial cotransmission over multiple generations is rare in this population. However, because polygenomic infections were identified based on the proportion of sites with non-unanimous reads, some of the infections classified as monogenomic may actually be polygenomic infections with extremely related parasite strains. This issue could be resolved by analyzing samples with higher read depth coverage. Because we were concerned about the loss of low frequency strains, our samples were directly sequenced from patient samples. This meant that the majority of generated reads aligned to the human genome. The genomes of parasites within some of these samples were only represented by 300 SNPs, which complicates the detection of sites with non-unanimous reads in highly related samples. Future studies could use selective whole genome amplification or hybrid selection to generate higher quality samples but will need to consider the potential for strain amplification bias.

A major implication of this work is that genetic epidemiology models can be improved by accounting for the genetic relatedness within polygenomic infections. The rates of superinfection and cotransmission may change depending on the transmission setting. In high transmission settings, genetic epidemiology models that simulate polygenomic infections as the result of superinfection may be sufficient, since superinfection is expected to be more common than cotransmission [15]. However, this assumption may be suspect, due to the observation of highly related haplotypes in polygenomic infections from high transmission settings [8], and cotransmission could still play a major role in these areas. In mid-low transmission settings, genetic epidemiology models should be adjusted to take into account the genetic relatedness of polygenomic infection owing to cotransmission, since superinfection will underestimate the genetic relatedness of polygenomic infections. Future studies are needed to quantify the relative rates of cotransmission and superinfection, but cotransmission can be incorporated into existing models by simulating the sampling of parental genotypes and sexual reproductive processes within the mosquito vector to determine the relatedness of the subsequent polygenomic infection. The explicit modeling of cotransmission connects the relatedness of polygenomic infections to the genetic composition of local parasite populations, allowing it to be affected by changes in transmission intensity and is applicable across any epidemiological setting.

The incorporation of related strains within polygenomic infection is important for understanding the genetic composition of parasite populations, particularly those in low transmission settings, since it can lead to differences in modeled expectations. Theoretical models of superinfection suggest that superinfection can greatly increase selection efficiency within the host [27] and can affect the fitness of drug-resistant parasites [28]. However, the presence of related strains within infections can alter these effects. For example, one study found that simulated infections composed of unrelated parasite strains can have different infection lengths compared to those of related strains [29]. Models that incorporate cotransmission should provide more accurate predictions, which will be helpful in malaria elimination activities to monitor transmission, assess the impact of interventions, and improve our understanding of the underlying biology and consequences on important traits, such as drug resistance, that threaten to undermine our elimination efforts.

Finally, the high prevalence of highly related polygenomic infections suggests that current methods for estimating COI can be improved. We previously published a method for estimating the COI of polygenomic infections based on a set of biallelic SNP markers [22]. Our method, known as COIL, assumes that polygenomic infections are composed of unrelated parasite strains, which we now know is not always the case in natural populations. Recognition that polygenomic infections can be composed of related parasite strains suggests that estimated COI levels could be reported as continuous rather than discrete values in settings where cotransmission is prevalent.

## Conclusions

To conclude, we find that models that simulate polygenomic infections through superinfection do not produce the high degree of relatedness observed within a set of 31 natural polygenomic infections collected from patients in Thiès, Senegal. The relatedness within these polygenomic infections suggests that cotransmission plays a major role in the persistence of polygenomic infections. Our data support the hypothesis that the cotransmission of genetically related parasite strains is common, and that this aspect of transmission should be incorporated into existing genetic epidemiology models. These findings have important implications for our understanding of malaria transmission, and potentially how important phenotypes like drug resistance that threaten to undermine malaria elimination activities may be promoted. As public health interventions drive parasite populations toward elimination, these models will play a critical role in helping us understand the changes in population structure associated with declining transmission rates and influencing the future of public health policy.

## Additional files

Additional file 1: Supplementary **Figures S1**–**S8** and Supplementary **Table S1.** (PDF 1415 kb)
Additional file 2: SRA BioProject accession numbers. (XLSX 35 kb)

## Abbreviations

COI: Complexity of infection
DBD: Different by descent
DNA: Deoxyribonucleic acid
EIR: Entomological inoculation rate
HMM: Hidden Markov model
IBD: Identical by descent
NMCP: National Malaria Control Programme
SNP: Single nucleotide polymorphism
