# Supplementary material for: Genetic relatedness analysis reveals the cotransmission of genetically related Plasmodium falciparum parasites in Thiès, Senegal
Source: Genome Med. 2017 Jan 24;9:5. doi: 10.1186/s13073-017-0398-0 (PMC5260019; doi:10.1186/s13073-017-0398-0)
Supplement: Additional file 1: — Supplementary Figures S1–S8 and Supplementary Table S1. (PDF 1415 kb) [file 13073_2017_398_MOESM1_ESM.pdf]

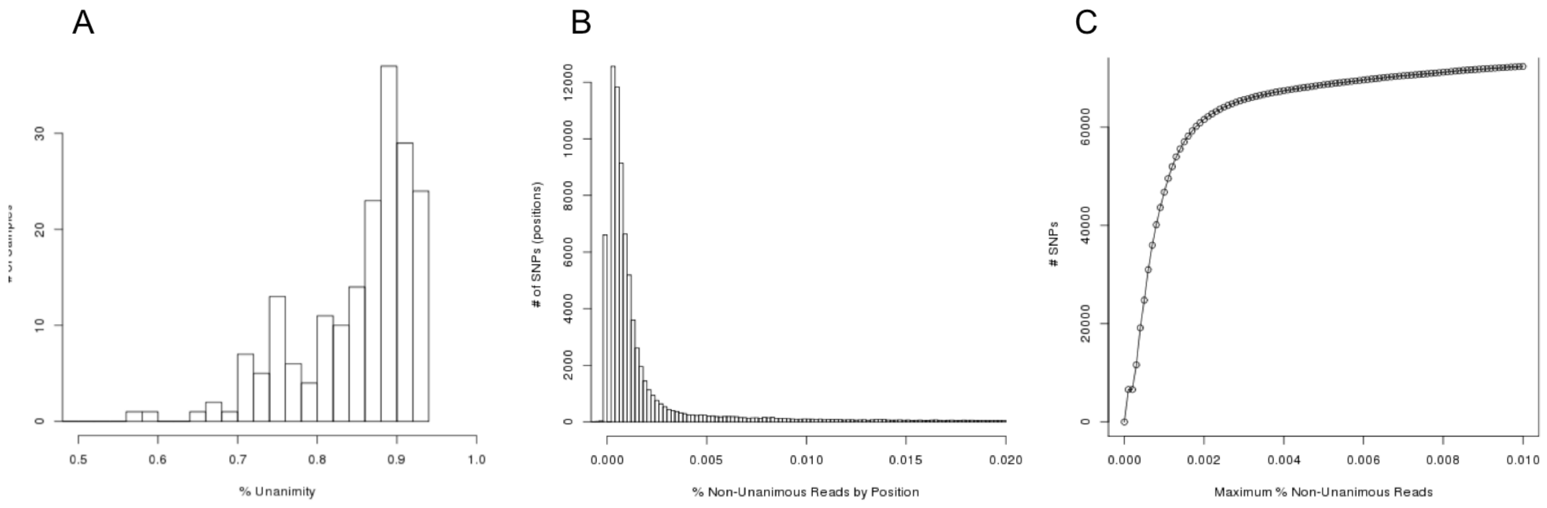

Figure S1. Developing a trusted SNP set. A) Histogram of allele balance across our set of preliminary trusted SNPs by sample. The preliminary SNP set consisted of 440,000 SNPs identified as having a non-unanimous pileup across 190 Senegal samples. An initial cut off of 80% was used to identify a set of putatively monogenomic infections. **B)** The percent non-unanimous reads across all the 440,000 SNPs in the 56 randomly chosen putative monogenomic infections. **C)** Cumulative density plot of the maximum percent of non-unanimous reads at each of the 440,000 sites. The read pileup over most sites has less than 0.2% of their reads with an alternative allele.

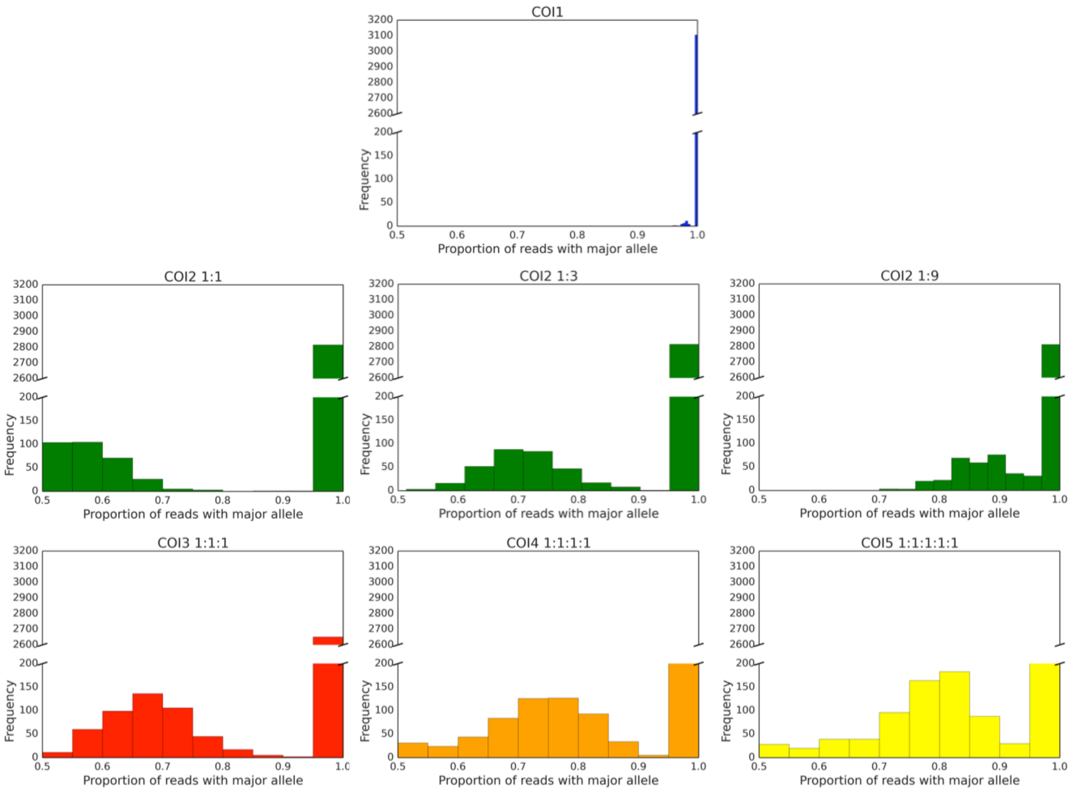

Figure S2. Histogram of proportions of reads supporting the major read

Histograms showing the proportions of reads supporting the major read over each of the 3132 trusted SNP sites for a variety of lab-generated mixtures. Sites where the proportion is equal to one indicates a unanimous read pileup. Sites where the proportion is 0.6 indicates that 60% of the reads show support for the major variant present in the read pileup while 40% if the reads show support for the minor variant. Blue histogram represents the lab mixture where COI = 1, green COI = 2, red COI = 3, orange COI = 4, and yellow COI = 5.

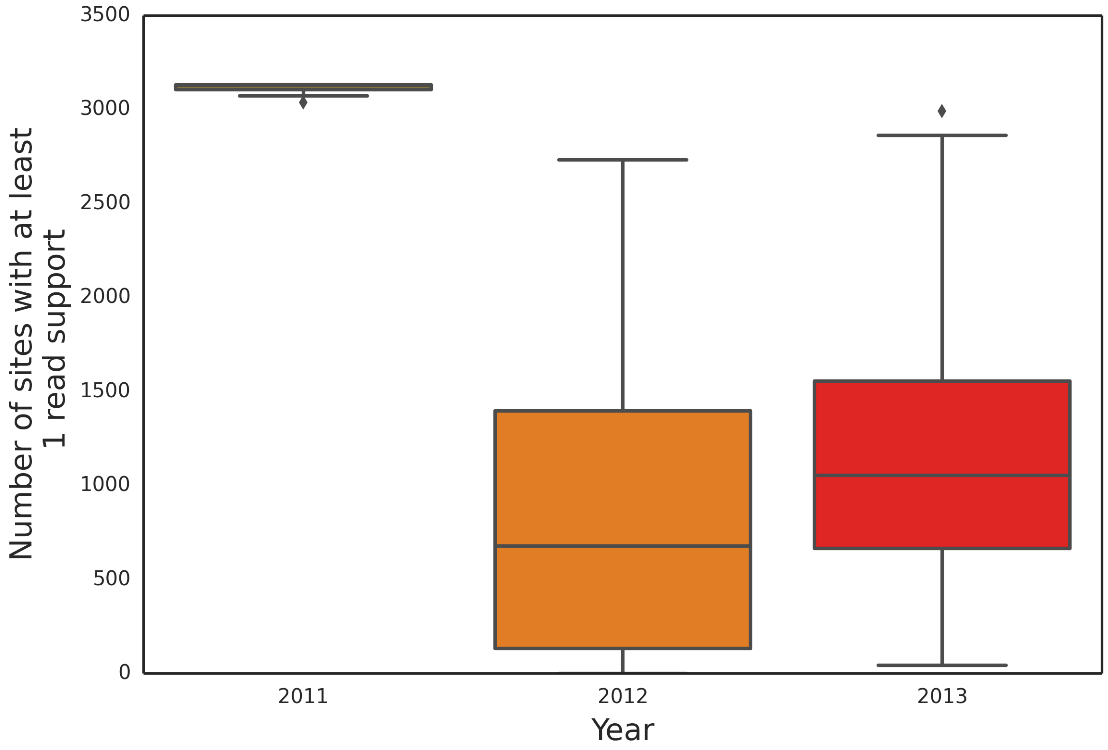

Figure S3. Trusted SNP set coverage for all 111 samples collected from 2011-2013.

Boxplots showing the number of trusted SNP sites with at least 1 read support. Each dot represents a sample collected from 2011-2013. Samples collected from 2011 had the most number of trusted SNP sites represented, while samples collected from 2012-2013 had a broad range in the number of represented sites.

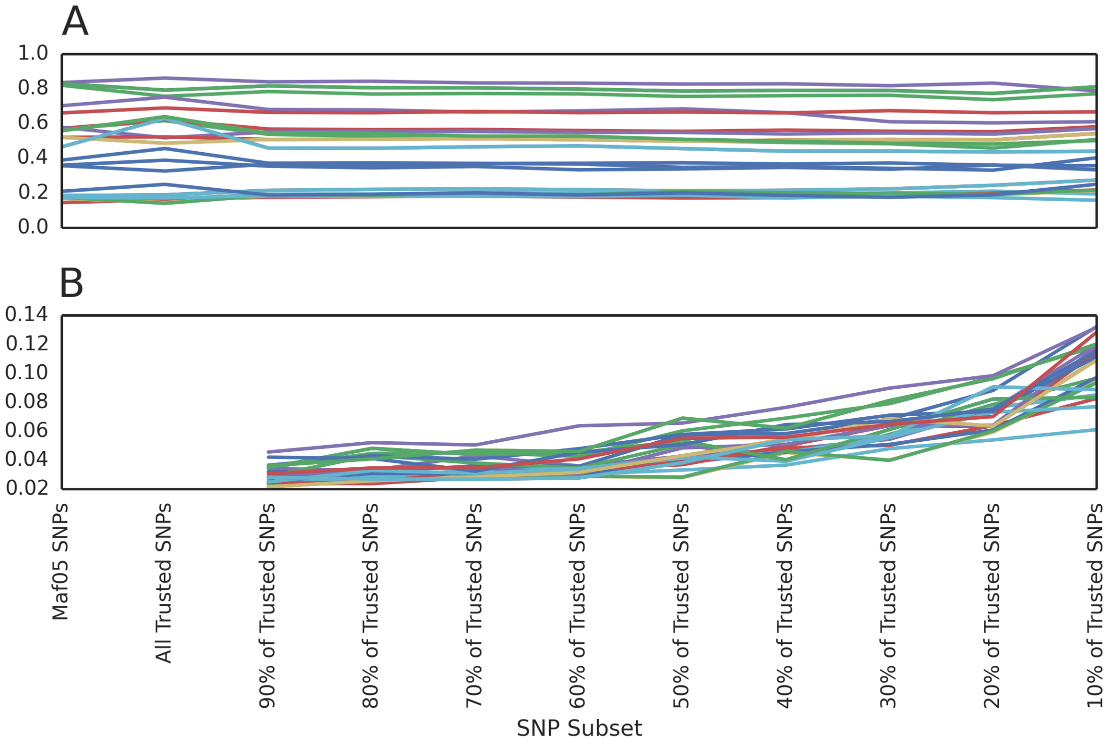

Figure S4. Sensitivity of HMM to different SNP subsets.

A) Mean relatedness estimates across different SNP subsets. For the genome-wide SNP set and the trusted SNP set, the mean is equal to the observed relatedness. For all other SNP sets, the mean represents the average relatedness across 40 randomly generated subsets. Each line represents a particular pairwise comparison

B) Standard deviation of relatedness estimates across different SNP subsets. For the genome-wide SNP set and the full trusted SNP set, we cannot calculate a standard deviation and are thus not plotted. For all other SNP sets, we calculated the standard deviation across 40 randomly generated subsets. Each line represents a particular pairwise comparison.

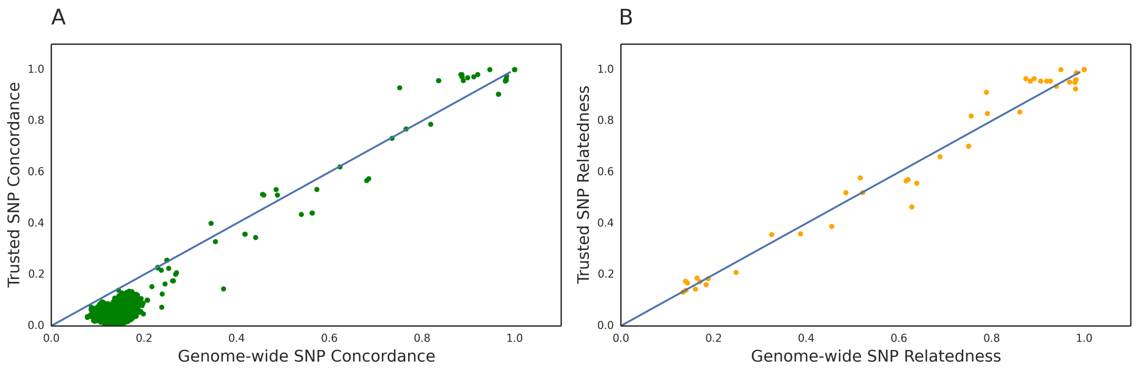

Figure S5. 3132 trusted SNP set vs genome-wide SNP set

Scatterplots representing the concordance (**A**) and relatedness (**B**) calculating using a set of 3132 trusted SNPs or the a set of 14,197 genome-wide SNPs. Concordance is the percent similarity at minor allele sites while relatedness is the proportion of the genome that is IBD. Individual points represent individual sample pairs. The blue diagonal line represents the 1-1 expectation.

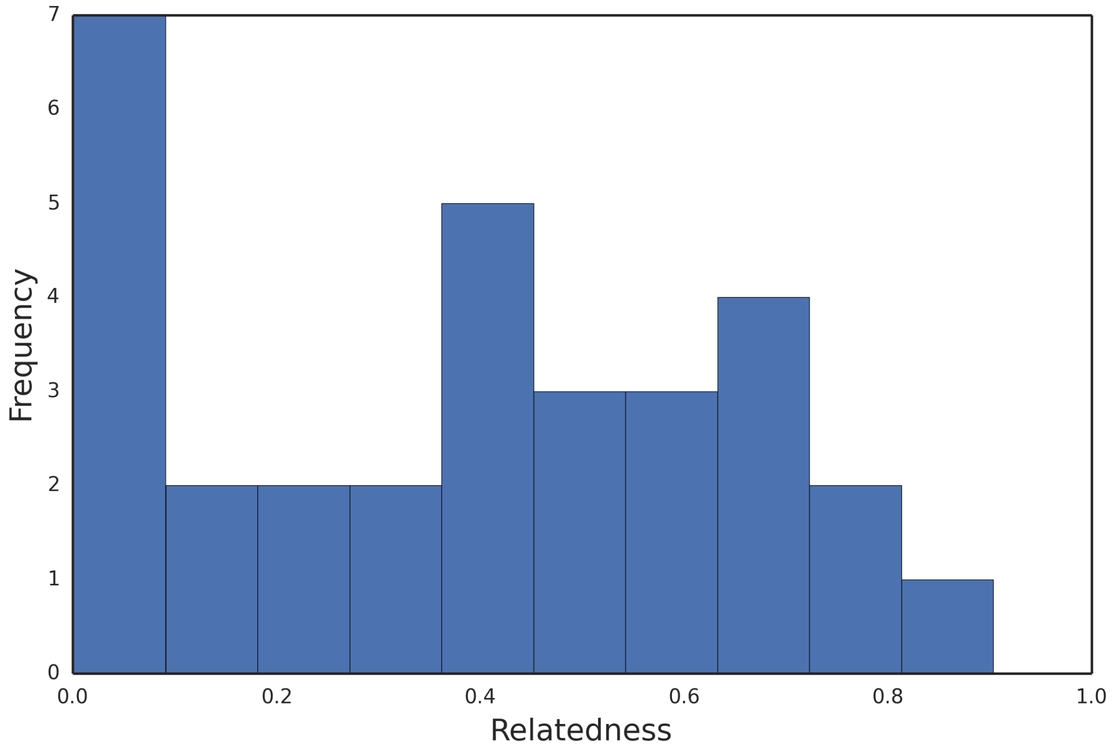

Figure S6. Histogram of relatedness within polygenomic infections

Histogram of jackknife estimates of the mean relatedness within 31 polygenomic infections collected from patients in Senegal from 2011-2013.

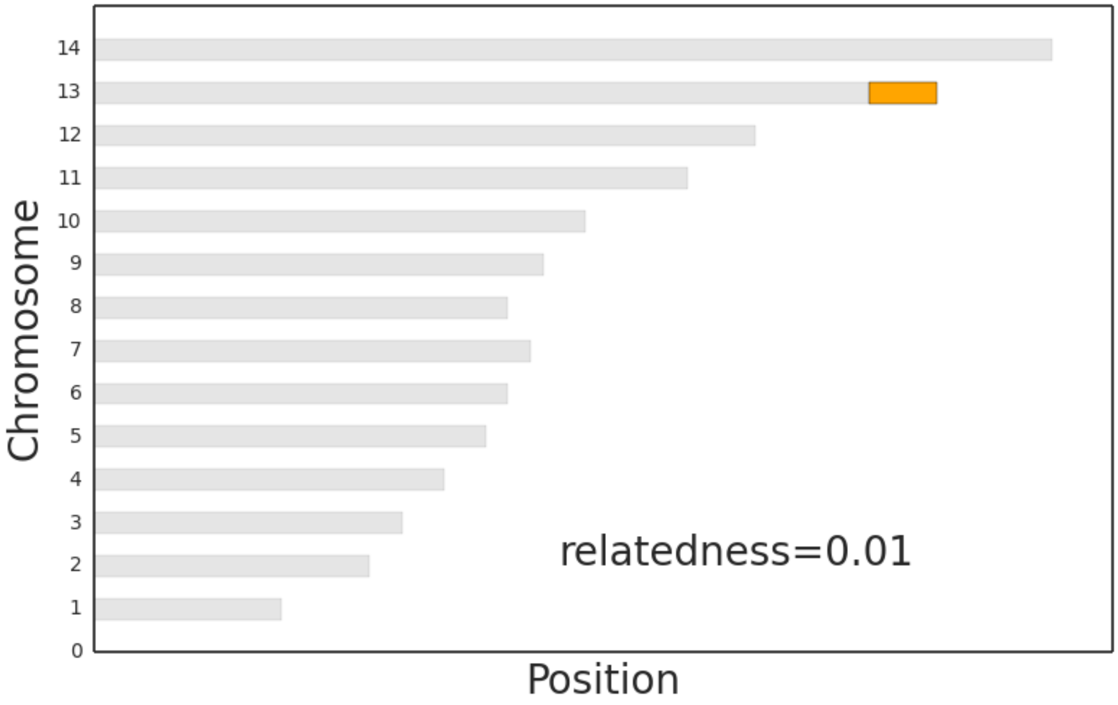

Figure S7. IBD map of the monogenomic strains related to SenT009.11

Orange represents the section of the genome that is IBD while grey represents the section of the genome that is not IBD.

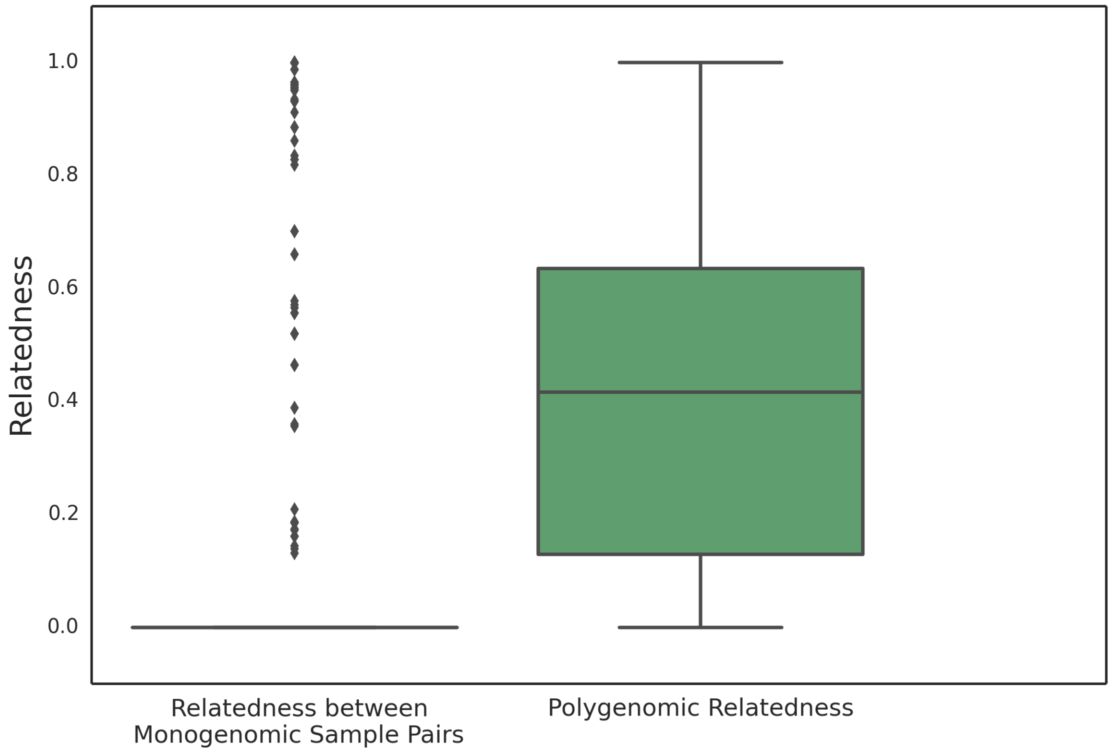

Figure S8. Naive resampling relatedness distributions

Boxplots of the relatedness between monogenomic sample pairs and the observed polygenomic infections. The distribution of relatedness among monogenomic infections is highly skewed, with 99% of the data having a relatedness of 0. The mean relatedness of this distribution is 0.07. The distribution of relatedness within polygenomic infections is much less skewed, with a mean relatedness of 0.4.

ID	Combination	Mixture
**COI5**	ABCDE	20:20:20:20:20
**COI4**	ABCD	25:25:25:25
**COI3a**	CDE	33:33:33
**COI3b**	CDE	40:20:40
**COI3C**	CDE	45:10:45
**COI3d**	CDE	49:02:49
**COI2a**	AB	50:50
**COI2b**	AB	75:25
**COI2c**	AB	90:10
**COI2d**	AB	95:05
**COI2e**	BC	50:50
**COI2f**	BC	75:25
**COI2g**	BC	90:10
**COI2h**	BC	95:05
**COI1A**	A	100
**COI1B**	B	100
**COI1C**	C	100
**COI1D**	D	100
**COI1E**	E	100

Table S1

Table of the strain composition and ratios of the lab-generated strain mixtures. A = SenT148.2009, B = SenT111.2009, C = SenT165.2009, D = SenT033.2009, and E = SenT015.2009. Sample concentrations were determined by nanodrop (Thermo).The mixtures of genomic DNA were made at final concentrations of 5ng/ul.
